# The Mad Stone

**Published:** 1876-12

**Authors:** 


The Mad Stone.—The following account of the effects of the "Mad Stone," as it is called, was lately published in the Philadelphia *Medical and Surgical Reporter*, and furnished to that paper for publication by M. P. Sigworth, M.D., of Paris, Iowa. The editor says that the writer is a regular physician in good standing. Who, it may be asked, can undertake to say there is not truth in the narative? It is strange, but not necessarily false. In the *Circular* for 1866, on pages 236 and 258, are two separate articles on this subject, and in the volume of 1870, on page 47, is another, all from different sources, that testify to similar wonderful absorbing and neutralizing powers of this stone. It may indeed be all imagination, fallacy or deception; but at any rate the claims for it, without preconcert of purpose, made by various individuals, make the "Mad Stone" not unworthy of scientific investigation. There is at least a second stone in Iowa.

Writes Dr. Sigworth: "The inquiry in your columns, in reference to the 'mad stone,' induces me to describe to you one now in this town. It is owned by a Mr. T. Evans, who received it as an heirloom from his ancestors.

"Having practiced medicine in this place for eleven years,

I have had abundant opportunities of seeing many cases cured by it, and of witnessing its beneficial effects on hydrophobia and snake bites. The mode of application is to scarify the skin and apply the stone; if there is any poison it will immediately adhere, so that it cannot be detached without great pain until it is full, and then it drops off. It will adhere from twenty minutes to one hour, when it will change to a green color. It is then put in warm milk and water, which it also changes to a green color. The first applications seem to cause a great deal of pain. Two weeks ago it adhered seventy-eight times in a case of hydrophobia. The case was of a man who had been bitten on the arm by a dog, not supposed to be mad, some six months ago. When he arrived here he had spasms about every fifteen minutes, violent in nature. The first application seemed to quiet him, and in twelve hours the spasms ceased, the man became rational, and slept for the first time in four days; in forty-two hours he was entirely relieved, the stone not adhering.

"The stone is of a slate or light color, porous, and will weigh from one-half to one ounce."

